# Physicochemical Properties of Sugarcane Cultivars Affected Life History and Population Growth Parameters of *Sesamia nonagrioides* (Lefebvre) (Lepidoptera: Noctuidae)

**DOI:** 10.3390/insects13100901

**Published:** 2022-10-03

**Authors:** Seyedeh Atefeh Mortazavi Malekshah, Bahram Naseri, Hossein Ranjbar Aghdam, Jabraeil Razmjou, Seyed Ali Asghar Fathi, Asgar Ebadollahi, Tanasak Changbunjong

**Affiliations:** 1 Department of Plant Protection, Faculty of Agriculture and Natural Resources, University of Mohaghegh Ardabili, Ardabil 5619911367, Iran; 2 Iranian Research Institute of Plant Protection, Agricultural Research, Education and Extension Organization (AREEO), Tehran 1985813111, Iran; 3 Department of Plant Sciences, Moghan College of Agriculture and Natural Resources, University of Mohaghegh Ardabili, Ardabil 5697194781, Iran; 4 Department of Pre-Clinic and Applied Animal Science, Faculty of Veterinary Science, Mahidol University, Nakhon Pathom 73170, Thailand

**Keywords:** sugarcane stem borer, life table, sugarcane cultivar, oviposition preference, biochemical traits

## Abstract

**Simple Summary:**

The sugarcane stem borer, *Sesamia nonagrioides* (Lefebvre), is the most important pest of sugarcane in Iran and some other regions of the world. Variation in the resistance of six commercial sugarcane cultivars to *S. nonagrioides* was investigated using the oviposition preference, life history, and population growth parameters. Moreover, the physical and biochemical properties of the tested sugarcane cultivars were estimated to understand any possible correlation between the insect’s parameters and the physiochemical features of the cultivars tested. The physicochemical properties of sugarcane cultivars significantly affected *S. nonagrioides* oviposition behavior, life history, and population parameters. Based on the obtained results, the resistant cultivar, SP70-1143, could be recommended for cultivation in sugarcane fields where the risk of *S. nonagrioides* damage is usually high.

**Abstract:**

The use of resistant cultivars is an efficient management strategy against *S. nonagrioides*. The effects of different sugarcane cultivars, CP48-103, CP57-614, CP69-1062, CP73-21, SP70-1143, and IRC99-02 were evaluated on the oviposition preference (free-choice assay), life history, and life table parameters of *S. nonagrioides* at 27 ± 1 °C, 60 ± 5% RH and a photoperiod of 16: 8 (L: D) h. The longest and shortest developmental times were on cultivars SP70-1143 and CP48-103, respectively. The oviposition preference of *S. nonagrioides* was the highest on cultivars CP48-103 and CP69-1062, and negatively correlated with the shoot trichome density and shoot rind hardness of the cultivars. The highest intrinsic rate of increase of *S. nonagrioides* was on cultivar CP48-103 and the lowest was on cultivar SP70-1143. The shortest mean generation time was on CP48-103 and the longest was on SP70-1143. The results indicate that cultivars CP48-103 and CP69-1062 were susceptible, and cultivar SP70-1143 was partially resistant against *S. nonagrioides*. This information could be useful for developing integrated management programs of *S. nonagrioides*, such as the use of resistant cultivars to reduce the damage caused by this pest in sugarcane fields.

## 1. Introduction

The need for more food for the world’s growing population has increased the quantity and quality of agricultural crops and their maintenance. Therefore, in order to optimally manage agricultural products, we must have correct information about production facilities, production methods, and monitor the production process until consumption [1,2,3]. Sugarcane, *Saccharum officinarum* L. (Poaceae), an industrial multi-usage plant, is a major source of sugar and raw materials for agro-industries [4]. This plant is extremely vulnerable to insect pest attacks, including stem borers belonging to *Sesamia* spp. [4].

The sugarcane stem borer, *Sesamia nonagrioides* (Lefebvre) (Lepidoptera: Noctuidae) is one of the most dangerous insect pests on sugarcane, corn, sorghum, millet, rice, grasses, melon, asparagus, palms, and banana [5,6]. It has a wide distribution in the Khuzestan and Fars provinces of Iran, southern Europe, North Africa, and the Middle East [7,8,9]. The sugarcane stem borer is a devastating pest on all phenological stages of the sugarcane in Khuzestan province where it has up to five generations per year [9]. The feeding of *S. nonagrioides* larvae at the tillering stage of sugarcane results in dead hearts and causes severe damage, after the formation of internodes, by reduction in stalk weight and sugar quality [10]. Both the quality and quantity of sugar extracted from sugarcane plants can be decreased when they are severely attacked by *S. nonagrioides* [11,12,13].

Owing to the development of insect pests’ resistance to insecticides and the negative effects of insecticides on human health and the environment, the use of alternative methods of insect control is essential [14,15]. Host plant resistance is one of the suitable alternatives to insecticides in pest management programs both economically and environmentally [16,17,18]. Based on integrated pest management (IPM) programs, the use of sugarcane cultivars that are resistant to stem borers could be an efficient method, along with other controlling methods such as chemical, cultural, and biological control [10,19,20,21].

The quality of host plants can influence the life history, population growth, oviposition preference, and feeding performance of insect pests [22,23]. Plant physicochemical properties including primary and secondary metabolites, as well as trichome density, tissue hardness, and moisture content can determine the oviposition behavior and population performance of insect pests [24,25,26,27,28]. Therefore, study of these parameters, as resistance indices, could be useful in the identification of resistant cultivars for use in IPM programs [29,30,31,32].

Several studies have been conducted on the resistance mechanisms, biology, and life table parameters of *S. nonagrioides* on different host plants. For example, Ranjbar Aghdam and Kamali [33] studied the rearing of *Sesamia cretica* Lederer and *S. nonagrioides* on various host plants under laboratory conditions and showed that maize and sorghum are suitable hosts to *S. nonagrioides* and *S. cretica*, respectively. The resistance mechanisms of sugarcane cultivars to *Sesamia* spp. were investigated by Asgarianzadeh [34], who identified that the antixenosis and tolerance were more important than antibiosis in providing resistance. The effects of sugarcane and maize were assessed on the life table parameters of *S. nonagrioides*, and sugarcane was reported to be more susceptible than maize to this pest [35].

Due to the importance of sugarcane as a strategic agricultural crop in Iran and many other countries of the world [4], and the high economic damages of *S. nonagrioides* on it, the current study was conducted to estimate the oviposition preference, life history, and life table parameters of *S. nonagrioides* on six commercially cultivated cultivars of sugarcane in Iran. In addition, we assessed the associations between the physical and biochemical traits of examined cultivars and the pest’s life history and life table parameters. The findings of this investigation could be helpful in an IPM program of *S. nonagrioides*, such as the use of genetically engineered cultivars that are resistant to this pest.

## 2. Materials and Methods

The experiments were performed in a growth chamber set at 27 ± 1 °C, 60 ± 5% RH, and a photoperiod of 16:8 (L:D) h in the Iranian Research Institute of Plant Protection, Agricultural Research, Education and Extension Organization (AREEO), Tehran, Iran. We used a completely randomized design for all the experiments.

### 2.1. Host Plant

The cut stems of 6 sugarcane cultivars, CP48-103, CP57-614, CP69-1062, and CP73-21 (Canal Point USA); SP70-1143 (Sao Paulo, Brazil); and IRC99-02 (cross-made in Cuba and selected in Iran) were collected from the sugarcane cultivars bank of Sugarcane and Byproduct Development Company, Khuzestan, Iran (48°46′53.81″ E and 31°50′23.79″ N). These cultivars were selected based on commerciality, cultivated area, and their reaction to the stem borers under field conditions. Some sugarcane cultivars such as SP70-1143 and CP57-614 were cultivated in Matana Agricultural Research Station, Egypt, showing the importance of these cultivars around the world [36]. About 100 plants from each cultivar were cultivated under field conditions. Stems between the elongation phase and the ripening phase (250–300 days after planting)) were cut every week and kept at 4 °C until their use in the experiments.

### 2.2. Physical and Biochemical Properties of Sugarcane Cultivars

To examine the relationship between sugarcane cultivars and *S. nonagrioides* population parameters and oviposition preference, some physical-biochemical traits of sugarcane cultivars were evaluated as follows:

#### 2.2.1. Density of the Shoot Trichomes

Sugarcane shoots were selected randomly from 5 plants of each cultivar. The trichome density of each cultivar was estimated by counting the number of trichomes per cm^2^ of the sheath margin (the upper, middle, and lower parts of the shoots) using a stereomicroscope (SZX16, Olympus) [37].

#### 2.2.2. Moisture Content

The 5-cm cut stems of each cultivar (five replicates) were weighed based on the fresh weight (*FW*) using an electronic balance (A & D Co., LTD, Tokyo, Japan, d = 0.01 g), and kept in an oven set at 70 °C for 48 h. After reaching a constant weight, they were re-weighed (dry weight: *DW*), and the moisture content (%*MC*) in the stems of each cultivar was calculated as the following equation:

$$MC=\frac{FW-DW}{FW}\times 100$$

#### 2.2.3. Rind and Pith Hardness

Using a fruit hardness tester (LutronFR-5120, Taiwan, tip size: 3 mm), the rind hardness of stems and shoots, and the pith hardness of stems were measured. The stems rind hardness was measured by piercing the middle part of the third and fourth internodes (as a target internode for the penetration of the stem borer larvae) at 300 days after planting, and the penetrating point was registered in Newton (N) as a hardness index. The pith hardness was determined in the cross-section (5-cm pieces) of the same internodes. The shoot rind hardness was measured by punching the initial and middle parts of the sugarcane shoots, as the target site for *S. nonagrioides* oviposition. Each test was conducted for each cultivar in five replications.

#### 2.2.4. Determination of Silica

One gram of the pulverized sample of the stems and shoots of different sugarcane cultivars was mixed with 9 g of lithium tetraborate (weight ratio 1:9) into a platinum labware, and then the final mixture was melted at 900 °C and dissolved in concentrated nitric acid. Eventually, 2 mL of the solution was placed inside the set ICP-OES (Varian Vista MPX), and the amount of silica in the sample was measured based on the different wavelengths (250–288 nm) created on the metal in the raw material [38].

### 2.3. Determination of Primary and Secondary Metabolites

#### 2.3.1. Sample Preparation

All experiments were performed in five replicates per cultivar. The biochemical metabolites of sugarcane cultivars were measured using the extract of sugarcane stems. After washing the stems in water (five stems from each cultivar), they were dried at room temperature for 4 h and chopped using an electric grinder.

#### 2.3.2. Determination of Total Protein

The protein content of the sugarcane cultivars was evaluated according to Bradford [39] using bovine serum albumin (BSA) as a standard. About 0.1 g of the powdered stems was mixed with 100 mL of 100 mM phosphate buffer and immediately centrifuged (12,000× rpm at 4 °C for 15 min). Then, 100 μL of the resulting supernatant was taken and transferred to the test tubes containing 5 mL of Bradford reagent. The absorbance was measured at 595 nm.

#### 2.3.3. Determination of Carbohydrate

The carbohydrate content was measured using the Anthrone reagent [40]. A quantity of 0.5 g of the powdered stems was mixed with 5 mL ethanol 80% and centrifuged (4000× rpm at 4 °C for 15 min). Then, 0.1 mL of the obtained supernatant was added to 3 mL of Anthrone reagent, and the absorbance was read at 620 nm.

#### 2.3.4. Determination of Total Phenolics

The total phenolics were assessed according to the Folin–Ciocalteu method [41]. The mixture of 0.5 g of the powdered stems and 5 mL of methanol 80% was centrifuged (4000× rpm at 4 °C for 15 min). A total of 1 mL of the obtained supernatant was transferred to a test tube and its volume was increased to 50 mL by distilled water. Then, 0.25 mL of Folin–Ciocalteu and 1.25 mL of sodium carbonate 20% were added to the mixtures and mixed completely. The tubes were kept at room temperature in the dark for 40 min. The absorbance of each sample was measured at 725 nm using a spectrophotometer (Unico, UV/Vis2100, Fairfield, NJ, USA). The result was expressed as mg of gallic acid equivalent/100 g of fresh weight sample.

#### 2.3.5. Determination of Total Flavonoids

The total flavonoids were determined using the aluminum chloride colorimetric assay method [42]. A total of 1 mg of powdered stems was mixed with 1 mL of 2% methanolic solution of aluminum chloride, and stored at room temperature for 15 min. The absorbance was read at 430 nm. The flavonoid content was expressed as mg of quercetin equivalents (QE)/g of fresh sample.

#### 2.3.6. Determination of Tannins

The condensed tannin content in the stems was analyzed using the vanillin–HCL method [43]. About 0.5 mg of the powdered samples was mixed with 3 mL of vanillin (4%) (*w*/*v*, vanillin in methanol). Then, 1.5 mL of HCL was added to this extract, and after storage at room temperature in the dark for 15 min, the absorbance was measured at 500 nm. The tannin values of the samples were expressed as mg of catechin equivalent (CE)/100 g of fresh sample.

### 2.4. Rearing of Sesamia nonagrioides

The initial population of *S. nonagrioides* was established using about 10,000 eggs collected from sugarcane fields of Sugarcane and Byproducts Development Company, Khouzestan, Iran, in May 2019. The eggs were kept in 9 cm Petri dishes with a piece of wet cotton ball to prevent the eggs from desiccation. The hatched neonate larvae were reared on the cut stems of each sugarcane cultivar in transparent plastic containers (19 × 13 × 4 cm) with a hole covered by a cloth mesh for ventilation and kept in a growth chamber set at 27 ± 1 °C, 60 ± 5% RH, and a photoperiod of 16:8 (L:D) h. Every 2–3 days, the larvae were transferred to new containers with fresh-cut sugarcane stems. After 7–10 days, the larvae that were developed to the third instar were reared individually on 20-cm cut stems until pupation. Each 10 cut stems were placed in cylindrical plexiglass jars (25 × 17 cm, Venus Co., Tehran, Iran), which were covered at the top by a fine mesh net for ventilation. After pupation, the sex of individuals was distinguished based on the distance between the sexual hole and anus, which is longer in females than in males [44], and the pupae were sterilized using 5% sodium hypochlorite solution [33]. Male and female pupae were kept separately in disposable containers (7 cm diameter × 3 cm depth with a cap) and checked daily until adult emergence. After the emergence of the adult moths, 3–7 pairs of moths (with equal sex ratio) were transferred into cylindrical transparent plexiglass jars (17 cm diameter × 25 cm depth) for mating and oviposition. Each oviposition jar contained 3–5 sugarcane shoots to provide natural oviposition substrate and a piece of cotton ball soaked in 10% honey:water solution as a source of carbohydrate for feeding of the adult moths [33]. During the oviposition period, the eggs were gathered from the leaf sheath daily using a fine brush (No. 000), and the sugarcane shoots were replaced with new ones. The sterilization of the eggs was carried out using the procedure described by Ranjbar Aghdam and Kamali [33]. In order to nutritionally adapt the *S. nonagrioides* on each sugarcane cultivar, the insect was reared for two generations on each cultivar, and the eggs obtained from the third generation were used in the experiments.

### 2.5. Oviposition Preference of Sesamia nonagrioides

The oviposition preference (number of eggs laid) of *S. nonagrioides* was tested on six sugarcane cultivars according to the free-choice assay. Three detached sugarcane shoots (length 20 cm) from each cultivar were randomly arranged in wooden cages (80 × 80 × 60 cm) covered by a white mesh cloth. Five pairs of one-day-old male and female adults were released in the center of the cage, and after 72 h, the eggs laid under the leaf sheath were counted separately for each cultivar. This experiment was performed in five replications.

### 2.6. Life History and Survival of Sesamia nonagrioides

The life history and survival of *S. nonagrioides* were investigated using 200 eggs (<24 h old) as a cohort for each treatment (sugarcane cultivar), which were laid by the females reared on the same cultivar. All 200 eggs for each cultivar were transferred into sterilized glass Petri dishes (diameter 9 cm), and the number of hatched eggs and their incubation periods were recorded daily. Newly emerged larvae (<24 h old) were placed individually in transparent plastic containers (9 cm diameter × 5 cm depth) containing a piece of the sugarcane cut stem (3 cm diameter × 7 cm height). The cut stems of each cultivar were replaced by fresh ones every 3–4 days during the larval development until prepupal stage. The pre-pupae remained unchanged inside the stems until pupation. After pupation, the pupae were sexed and weighed using laboratory scales (Sartorius AG Germany GCA803S, *d* = 0.001 ct) at 24 h after pupation on each sugarcane cultivar and placed individually in new containers (7 cm diameter × 3 cm depth with a cap) until adult emergence. Larval and pupal containers were checked daily, and their duration and survival were recorded. The emerged adult moths were transferred to oviposition jars (17 cm diameter × 25 cm depth), and adult longevity and the number of eggs laid by each female (fecundity) were recorded daily. In order to determine egg hatching (fertility), the egg masses laid at different times were kept separately. Daily monitoring was continued until the death of the last female and male moths. The sex ratio was calculated as the following formula:

$$\mathrm{Sex}\;\mathrm{Ratio}=\frac{\mathrm{females}}{\mathrm{females}+\mathrm{males}}\times 100$$

### 2.7. Life Table Parameters of Sesamia nonagrioides

The life table (population growth) parameters of *S. nonagrioides* were assessed based on the age-stage, two-sex life table theory [45,46] using TWOSEX-MSChart software [47]. The age-specific survival rate (*l_x_*) was calculated as:

$$l_{x}=\sum_{j=1}^{k}s_{x}{}_{j}$$

where *k* is the number of stages and *s_xj_* is the probability that newly laid eggs will survive to age *x* and stage *j*. Age-specific fecundity (*m_x_*) was measured using the following formula:

$$m_{x}=\frac{\sum_{j=1}^{k}s_{x}{}_{j}f_{xj}}{\sum_{j=1}^{k}s_{xj}}$$

where *f_xj_* (age-stage-specific fecundity) is the number of hatched eggs that were produced by an adult female at age *x*. The intrinsic rate of increase (*r*) was calculated as the following formula:

$$1=\overset{\infty }{\underset{x=0}{\sum }}e^{-r\left(x+1\right)}l_{x}m_{x}$$

Other population growth parameters including the gross reproductive rate (*GRR*), net reproductive rate (*R*_0_), finite rate of increase (*λ*), and mean generation time (*T*) were calculated as *GRR*=$\overset{\infty }{\underset{x=0}{\sum }}mx$, *R*_0_=$\overset{\infty }{\underset{x=0}{\sum }}l_{x}m_{x}$, *λ*=*e^r^* and *T* = (ln *R*_0_)/*r* [48,49].

### 2.8. Statistical Analysis

The physicochemical traits data, the weights of pupae, and the oviposition preference of *S. nonagrioides* were examined for normality with a Kolmogorov–Smirnov test using SPSS version 16.0 [50], and all the data were normally distributed. These data were analyzed by a one-way analysis of variance (ANOVA), and the statistical differences among the treatments were compared using Tukey’s HSD test at a 5% probability level. The developmental time, adult pre-oviposition period (APOP: the time between the emergence of an adult female and the start of its oviposition), total pre-oviposition period (TPOP: the duration from egg to first oviposition), oviposition period, fecundity, and life table parameters were analyzed based on the age-stage, two-sex life table theory [45,46]. Additionally, the bootstrapping with 100,000 replications was used to estimate the variances and standard errors of life history and population parameters [51,52], and the paired bootstrap test was used to compare the means [53,54]. The relationship between the physicochemical features of the tested sugarcane cultivars and *S. nonagrioides* oviposition preference and population parameters was evaluated by Pearson correlation test using the average data of variables [55,56]. A dendrogram by Ward’s method was drawn based on the oviposition preference, life history, and life table parameters of the insect reared on examined sugarcane cultivars using SPSS version 22.0 (IBM, Chicago, IL, USA).

## 3. Results

### 3.1. Properties of Sugarcane Cultivars

The physical and biochemical features of sugarcane cultivars used for the feeding of *S. nonagrioides* are presented in Table 1. The highest trichome density in sugarcane shoot was observed in cultivar SP70-1143, whereas the lowest density was determined in cultivars CP48-103, CP69-1062, and IRC99-02 (*F* = 278.34; df = 5, 29; *p* < 0.001). The stem of cultivar SP70-1143 (66.91%) had the lowest moisture content compared with other cultivars (*F* = 352.57; df = 5, 29; *p* < 0.001). The results showed significant differences in the shoot rind hardness (F = 1176.21; df = 5, 29; *p* < 0.001) and stem rind hardness (*F* = 6020.53; df = 5, 29; *p* < 0.001) among the various sugarcane cultivars. The stem and shoot rind hardness indices were the highest in cultivar SP70-1143 and the lowest in cultivar CP48-103. Among the tested sugarcane cultivars, the highest value of stem pith hardness was recorded in cultivar SP70-1143 (*F* = 977.49; df = 5, 29; *p* < 0.001).

**Figure 1:**
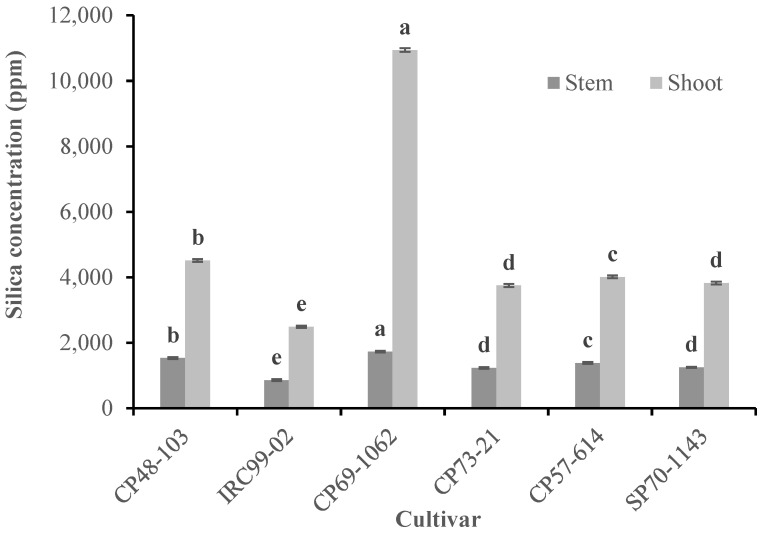
Mean (±SE) silica concentration (ppm) in the stem and shoot of tested sugarcane cultivars. Mean values followed by different letters are significantly different according to Tukey’s HSD test (*p* < 0.05).

The highest amount of silica was observed in the stem (*F* = 389.66; df = 5, 17; *p* < 0.001) and shoot (*F* = 13,837.68; df = 5, 17; *p* < 0.001) of cultivar CP69-1062, while the lowest amount was observed in the stem and shoot of cultivars CP73-21 and SP70-1143 (Figure 1).

The total protein content (*F* = 139.18; df = 5, 24; *p* < 0.001) significantly varied among the tested sugarcane cultivars; the lowest values were in cultivars CP48-103 and CP69-1062, and the highest value was in cultivar SP70-1143. The carbohydrate content in cultivar CP69-1062 was significantly higher than in the other cultivars (*F* = 87.27; df = 5, 29; *p* < 0.001). The condensed tannins content was the highest in cultivar SP70-1143, whereas the lowest was in CP48-103 (*F* = 94.82; df = 5, 29; *p* < 0.001). A significant difference in the total phenolics (*F* = 197.10; df = 5, 29; *p* < 0.001) and total flavonoids (*F* = 872.13; df = 5, 29; *p* < 0.001) was detected in different sugarcane cultivars; with higher content in cultivar SP70-1143 and lower content in cultivar CP48-103.

### 3.2. Oviposition Preference of Sesamia nonagrioides

**Figure 2:**
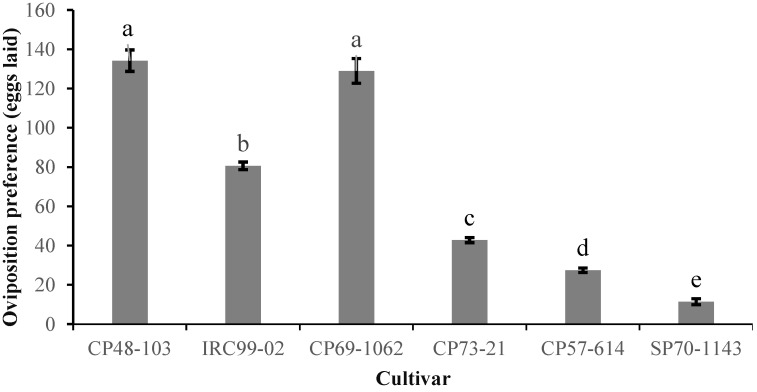
Mean (±SE) oviposition preference of *Sesamia nonagrioides* on different sugarcane cultivars. Mean values followed by different letters are significantly different according to Tukey’s HSD test (*p* < 0.05).

The free-choice oviposition preference of *S. nonagrioides* on the sugarcane cultivars is displayed in Figure 2. The maximum number of eggs laid was on cultivars CP48-103 and CP69-1062, and the minimum number was on cultivar SP70-1143 (*F* = 1043.05; df = 5, 29; *p* < 0.001).

### 3.3. Developmental Time and Survival of Sesamia nonagrioides

The outcomes of the effect of different sugarcane cultivars on the developmental time and survival (from egg to adult emergence) of *S. nonagrioides* are shown in Table 2. The average incubation period of *S. nonagrioides* on cultivar CP48-103 was significantly shorter than on the others (*p* < 0.05). The longest larval period was on cultivar SP70-1143 (50.09 ± 2.12 days), and the shortest was on cultivar CP48-103 (31.63 ± 1.02 days) (*p* < 0.05). No significant differences (*p* > 0.05) were observed for pre-pupal period among the sugarcane cultivars. The longest and shortest pupal period was seen on cultivars SP70-1143 (11.19 ± 0.07 days) and CP69-1062 (10.52 ± 0.05 days) (*p* < 0.05). The mean developmental time (from egg to adult emergence) on cultivar CP48-103 was about 25.50 days shorter than on cultivar SP70-1143 (*p* < 0.05). The highest immature (egg, larva, prepupa and pupa) survival was on cultivars CP48-103 (81.25 ± 3.07%) and CP69-1062 (76.25 ± 3.36%), and the lowest on SP70-1143 (19.34 ± 2.56%).

### 3.4. Weight of Male and Female Pupae

**Figure 3:**
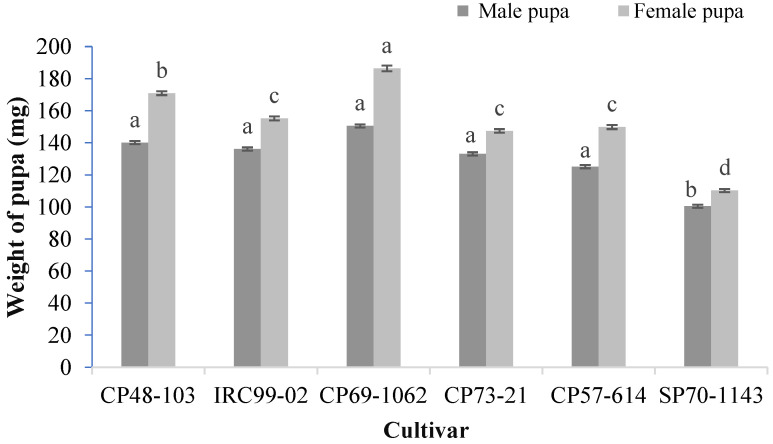
Mean (±SE) weight of pupal stage (mg) of *Sesamia nonagrioides* on examined sugarcane cultivars. Mean values followed by different letters are significantly different according to Tukey’s HSD test (*p* < 0.05).

Figure 3 shows the weight of the male and female pupae of *S. nonagrioides* on the examined sugarcane cultivars. The mean weight of the female pupae significantly varied from 110.2 mg on cultivar SP70-1143 to 186.4 mg on cultivar CP69-1062 (*F* = 78.06; df = 5, 164; *p* < 0.001). Furthermore, the male pupal weight on cultivar SP70-1143 (100.4 mg) was significantly lower than the other cultivars (*F* = 5.37; df = 5, 179; *p* < 0.001).

### 3.5. Longevity and Reproductive Variables of Sesamia nonagrioides

Adult longevity showed significant differences on the examined sugarcane cultivars (*p* < 0.05). The longevity of both male and female adults of *S. nonagrioides* was shortest on cultivar SP70-1143, while the longest female longevity was observed on cultivars CP48-103 and CP69-1062 (Table 3). The pre-oviposition and oviposition periods, fecundity and fertility of *S. nonagrioides* on the tested cultivars are presented in Table 3. Various sugarcane cultivars had a significant influence on the adult pre-oviposition period (APOP) and total pre-oviposition period (TPOP) of *S. nonagrioides*. The longest and shortest APOP was on cultivars SP70-1143 and CP69-1062, respectively (*p* < 0.05). The longest TPOP was observed on cultivar SP70-1143 and the shortest was on cultivars CP69-1062 and CP48-103 (*p* < 0.05). Moreover, the longest oviposition period was on cultivars CP48-103 and CP69-1062, and the shortest was on cultivar SP70-1143 (*p* < 0.05). The fecundity and fertility of *S. nonagrioides* were the highest on cultivars CP48-103 and CP69-1062, and the lowest on cultivar SP70-1143 (*p* < 0.05).

### 3.6. Survival Rate and Fecundity Curves

**Figure 4:**
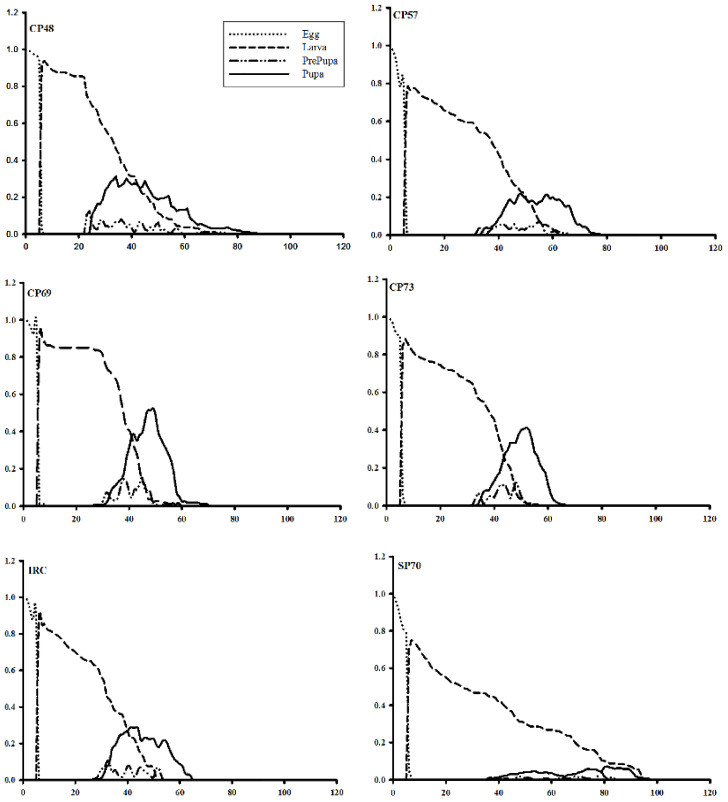

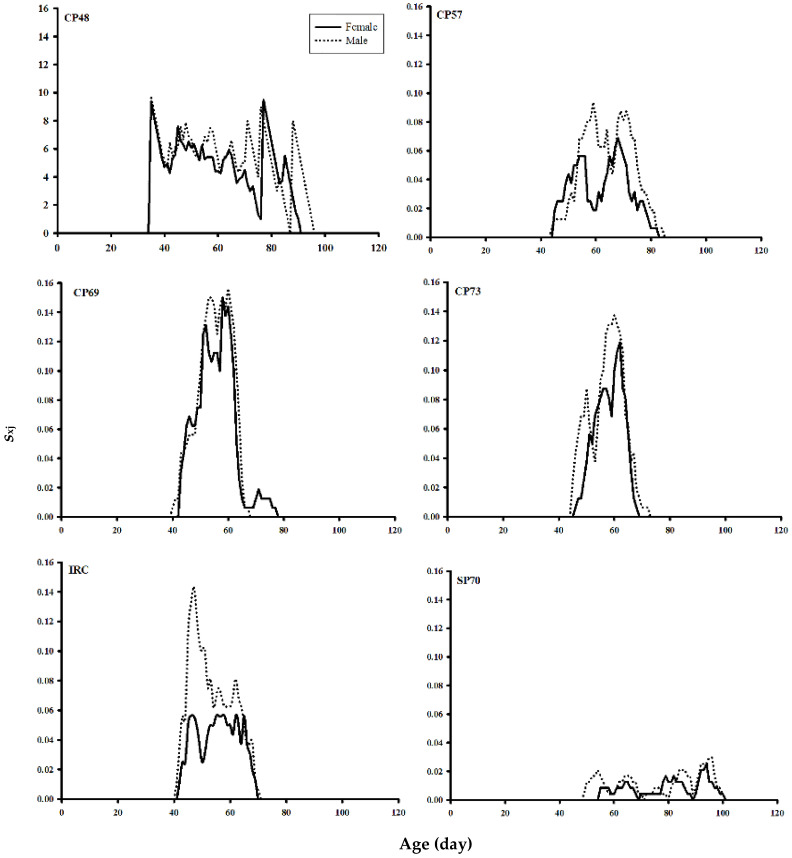
Age-stage survival rate (*s_xj_*) of *Sesamia nonagrioides* on examined sugarcane cultivars.

The age-stage-specific survival rate (***s***_*xj*_) of *S. nonagrioides* on various sugarcane cultivars is presented in Figure 4. The survival rates of *S. nonagrioides* at similar ages and developmental stages on CP48-103 and CP69-1062 were higher than on the other tested cultivars. Furthermore, the survival curves of male and female adults were further outspread on these cultivars, indicating an increase in oviposition period and the insect population on these cultivars.

**Figure 5:**
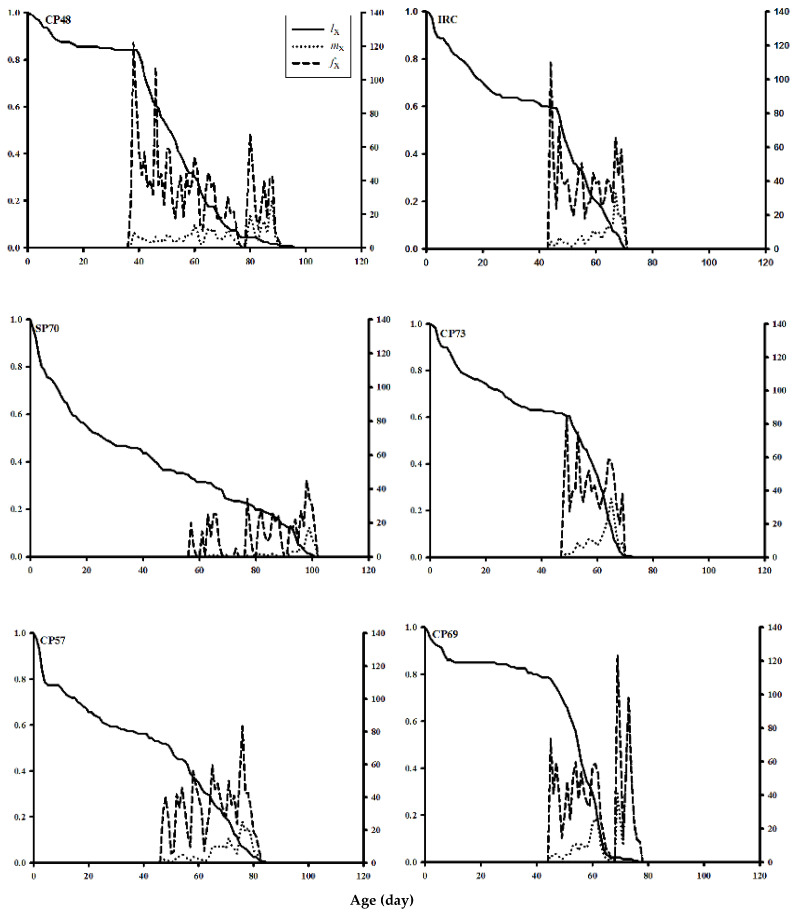
Age-specific survival rate (*l_x_*), age-specific fecundity (*m_x_*), and age-stage-specific fecundity (*f_xj_*) of *Sesamia nonagrioides* on examined sugarcane cultivars.

The age-specific survival rate (*l_x_*), age-specific fecundity (*m_x_*), and the age-stage-specific fecundity (*f_xj_*) of *S. nonagrioides* on various cultivars are plotted in Figure 5. The convexity of the *l_x_* curve was highest on cultivars CP48-103 and CP69-103, while the highest concavity of this curve was seen on cultivar SP70-1143. The *m*_x_ curve indicated that the highest number of eggs laid occurred on day 72 (98 eggs per individual per day) on CP69-1062. However, the reproduction of female moths performed much earlier on the CP48-103 cultivar (on the thirty-sixth day) than the other cultivars. Moreover, females lived longer on this cultivar. The highest *f_xj_* of *S. nonagrioides* on CP48-103, CP69-1062, CP73-21, IRC99-02, CP57-614, and SP70-1143 was122, 123, 85, 110, 83, and 45 eggs per female per day, respectively. These curves showed that the survival and fecundity rates, as well as oviposition period, were higher on CP69-1062 and CP48-103 cultivars than the other cultivars.

The sex ratio of *S. nonagrioides* was also affected by the host plant cultivars. The results show that the values of sex ratio were 55.74, 51.64, 48.42, 44.21, 46.75, and 42.01% on cultivars CP48-103, CP69-1062, CP73-21, IRC99-02, CP57-614, and SP70-1143, respectively.

### 3.7. Life Table Parameters of Sesamia nonagrioides

The population growth parameters of *S. nonagrioides* reared on different sugarcane cultivars are given in Table 4. There were significant differences in all computed population parameters on the examined cultivars. The lowest and highest gross reproductive rate (*GRR*) was observed on cultivars SP70-1143 and CP69-1062, respectively (*p* < 0.05). The highest net reproductive rate (*R*_0_) was observed on cultivars CP48-103 and CP69-1062 and the lowest on cultivar SP70-1143 (*p* < 0.05). The intrinsic rate of increase (*r*) and finite rate of increase (*λ*) were the highest when the insect was reared on cultivar CP48-103, and the lowest when it was reared on cultivar SP70-1143 (*p* < 0.05). Among sugarcane cultivars, the mean generation time (*T*) significantly differed from 47.06 days on cultivar CP48-103 to 82.63 days on cultivar SP70-1143 (*p* < 0.05).

### 3.8. Correlation Analysis

Table 5 indicates the values of correlation coefficients between *S. nonagrioides* life history, life table parameters, and oviposition preference and different physicochemical characteristics of the tested sugarcane cultivars. The life history and life table parameters of *S. nonagrioides* were not significantly correlated with the carbohydrate content of the cultivars. Significant positive correlations were found between the developmental time and condensed tannins, total phenolics, and flavonoids content of the sugarcane cultivars. The survival rate of immature stages, fecundity, weight of pupae (male and female), oviposition preference, *R*_0_, *r*, and *λ* of *S. nonagrioides* were negatively correlated with the protein content, tannins content, total phenolics, and flavonoids content of the cultivars. In contrast, the *T* values of *S. nonagrioides* were positively correlated with the protein content, tannins content, total phenolics, and flavonoids content of the sugarcane cultivars.

There was a significant negative correlation between *R*_0_ values with the trichome density and shoot hardness index of sugarcane cultivars. The oviposition preference, immature survival, *r* and *λ* values of *S. nonagrioides* were negatively correlated with shoot trichome density and the hardness index of the shoot and stem, whereas these variables were positively correlated with the moisture content of the cultivars. Positive correlations were discovered between the developmental time and *T* values of *S. nonagrioides* with shoot trichome density and the hardness index of the shoot and stem, while there were negative correlations between moisture content and *S. nonagrioides* developmental time and *T* values (Table 5).

Significant correlations were found for the weight of male and female pupae with the life table variables and fecundity of *S. nonagrioides* (Table 6). There were positive correlations between the pupal weight and fecundity, *R*_0_, *r* and *λ* of *S. nonagrioides*, whereas *T* value was negatively correlated with pupal weight.

### 3.9. Cluster Analysis

**Figure 6:**
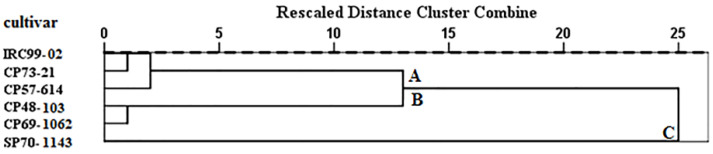
Dendrogram of sugarcane cultivars classified based on the oviposition preference and population parameters of *Sesamia nonagrioides* fed with these cultivars (Ward’s method).

A dendrogram based on the oviposition preference and population growth parameters of *S. nonagrioides* on various sugarcane cultivars is shown in Figure 6. The tested sugarcane cultivars were grouped into three clusters: A (cultivars IRC99-02, CP73-21 and CP57-614); B (cultivars CP48-103 and CP69-1062); and C (cultivar SP70-1143).

## 4. Discussion

The findings of this research revealed significant effects of the sugarcane cultivars on the oviposition preference, growth, development, survival, and reproduction of *S. nonagrioides.* In this study, the plant secondary metabolites, moisture content, shoot trichome density, and shoot hardness had a notable effect on the oviposition choice of *S. nonagrioides* because these variables were significantly correlated with the oviposition preference. Previous investigations indicated that trichome density [37,57], concentrations of secondary metabolites [32], and tissue hardness [58,59] significantly affected host selection by ovipositing female insects. The outcomes of the oviposition preference and population performance of *S. nonagrioides* demonstrated the suitability of cultivars CP48-103 and CP69-1062 for the offspring development and population increase of the pest. This agrees with the preference–performance theory proposed by Jaenike [60], who expressed that ovipositing female insects choose the best-quality host plants for the survival and optimal growth of their progeny.

The population dynamics of herbivorous insects can be influenced by environmental factors, natural enemies, and insecticides [61,62,63,64,65], as well as host plants [66,67,68,69]. The development of resistant cultivars serves as an effective approach in IPM to reduce losses due to insect attacks [70,71]. In the present study, *S. nonagrioides* developed rather quickly on cultivar CP48-103, demonstrating its better nutritive quality than the other tested cultivars. Differences in the duration of immature stages of herbivorous insects on various plant cultivars might be relevant to the nutritional quality and quantity or biochemical attributes of the host plants [72,73]. Moreover, in this study, associations between the hardness index and moisture content of the tested cultivars and the developmental time of *S. nonagrioides* showed that these physical traits could be important factors responsible for the development of immature stages. In some cultivars of sugarcane, thickened stems prevent the penetration of larvae or slow their penetration into the stem by increasing the layers of epidermal cells [59]. The shortest larval period of *S. nonagrioides* on cultivar CP48-103 may be related to lower concentrations of secondary metabolites such as tannins, phenolics, and flavonoids in this cultivar. Moreover, it may be due to the lower hardness index of rind and stem and the higher moisture content of this cultivar, which leads to the easier penetration and feeding of larvae inside the stem. Positive correlations between the developmental time of *S. nonagrioides* and the concentration of condensed tannins, total phenolics and flavonoids, and hardness index of tested cultivars indicated that, among physicochemical features, these factors play the most important role in the developmental rate of *S. nonagrioides*. Furthermore, developmental time was negatively correlated with the amount of moisture in the stems of sugarcane cultivars, which revealed its role in the development of the immature stages of this pest. The slow development of the immature stages of *S. nonagrioides* on cultivar SP70-1143 can be attributed to the higher concentrations of secondary metabolites, lower moisture content, and higher hardness index in this cultivar. In our study, the larval and pupal periods of *S. nonagrioides* on the tested cultivars were longer than those reported by Ranjbar Aghdam [74] and Sedighi [35]. These discrepancies can be due to the differences in the genetic pattern of plant cultivars and the pest, as well as the experimental conditions.

Body weight is one of the major biological indices of the insect population, which is correlated to the type of diet consumed [75]. The high pupal weight of the individuals reared on cultivar CP69-1062 demonstrated that this cultivar is more favorable than the others. In this study, negative correlations between pupal weight and the concentration of tannins, total phenolics, and flavonoids suggested that the larval and pupal growth are affected by the quality of food consumed or the amount of secondary compounds [29,76]. Moreover, the nutritional effects of unsuitable cultivars are reflected in weight of the pupae [32,77]. Therefore, the high pupal weight on cultivar CP69-1062 might be related to the low contents of tannins, total phenolics, and flavonoids in this cultivar. No significant correlation between pupal weight and the carbohydrate content of sugarcane cultivars points to the more important effect of secondary compounds than carbohydrate on the growth of pupae [32,77]. According to the results of this study, the females reared on cultivars CP69-1062 and CP48-103 had higher longevity and fecundity. Therefore, these cultivars were suitable hosts for the growth and development of the pest, which led to the emergence of adults with higher potential reproduction. These results can be related to the lower concentration of tannins, total phenolics, and flavonoids in these cultivars than the other cultivars. This is confirmed by the negative correlation observed between *S. nonagrioides* female longevity and fecundity and the secondary metabolites content of the tested cultivars. Furthermore, the lowest female longevity and fecundity of *S. nonagrioides* on cultivar SP70-1143 might be attributed to the high contents of tannins, total phenolics, and flavonoids in this cultivar, which revealed its poor nutritional quality for this pest. In the other words, the immature survival and development of *S. nonagrioides* on cultivar SP70-1143 are negatively influenced by the secondary compounds, leading to reduced adult longevity and reproductive capacity. The pupal weight is one of the main indices of fecundity performance (number of eggs laid) in female adults. The high pupal weight expresses the better nutrition of the insect in the larval stage and consequently increases adult longevity and reproductive potential. Cultivars CP69-1062 and CP48-103 had suitable conditions for the growth and nutrition of *S. nonagrioides* larvae, which led to the increase in the pupal weight, fecundity, and fertility of this pest. Moreover, no significant correlation between physical traits and adult longevity and fecundity suggested that these traits have no significant effect on the adult life history parameters. The longest immature period and lowest pupal weight of *S. nonagrioides* on cultivar SP70-1143 can be attributable to the low amount of moisture, high trichome density, and shoot and stem hardness in this cultivar. Previously, it has been reported that the quality and quantity of nutrients in the host plants are major factors influencing the adult longevity and reproduction of herbivorous insects [28,32,78]. Therefore, the quality of food and the presence of secondary metabolites in host plants are key factors affecting the survival and growth rate of herbivorous insects, which in turn will affect the reproductive potential of the adults [79].

The highest survival rate and shortest developmental time of *S. nonagrioides* on cultivar CP48-103 are likely due to its better nutritional quality, which results in increased values of the growth index [80,81]. Differences in nutrient quality or physicochemical characteristics among sugarcane cultivars can influence *S. nonagrioides* larval growth. In this study, cultivar CP48-103 showed low levels of tannins, total phenolics, and flavonoids, low hardness of the shoot and stem, low density of trichomes, and high amount of moisture. Hence, it is a suitable cultivar for the development and survival of this pest. Moreover, the high mortality of the pest on cultivars SP70-1143, CP73-21, and CP57-614 may be related to the high shoot hardness, and high amounts of tannins, phenolics, and flavonoids in these cultivars. Although no significant correlations were found, in our study, between *S. nonagrioides* population parameters and the tested properties of the sugarcane cultivars, previous works showed that in cultivars with high silica content, the inner surface of the mandibles of *S. nonagrioides* larvae could be damaged during the larval feeding process due to the increased hardness of stem tissues, and many of these larvae die due to decreased digestibility or starvation [82,83,84]. Sugarcane cultivars can absorb silica and amass it in the plant leaves and stalks, leading to an increase in plant resistance to pests [84,85].

The intrinsic rate of increase (*r*) is an important index for expressing the level of plant resistance to insects, which is greatly affected by the nutritional quality of host plant [86]. The highest and lowest *r* values were on cultivars CP48-103 and SP70-1143, respectively; hence, these cultivars were the most susceptible and resistant, respectively. The sugarcane stem borers reared on cultivar CP48-103 had greater survival, fecundity, and fertility, and shorter developmental time, which resulted in higher *r* value. Furthermore, the increase in the *R*_0_ and *GRR* values of *S. nonagrioides* fed on cultivars CP48-103 and CP69-1062 may be attributed to the better nutritive quality of these cultivars than the other tested cultivars (e.g., low tannins content, total phenolics, flavonoids, low shoot and stem stiffness, and high moisture content in these two cultivars). Furthermore, the lowest values of *r*, *R*_0_, *GRR*, and *λ* of *S. nonagrioides* on cultivar SP70-1143 can be related to high amounts of tannins, total phenolics, flavonoids, high shoot and stem stiffness, and low moisture content in this cultivar. Therefore, the mentioned physicochemical characteristics can be the important resistance factors of cultivar SP70-1143 to this pest. In our study, there is a negative correlation between the *r*, *R*_0_, *GRR*, and *λ* of *S. nonagrioides* and secondary metabolites content, trichome density, and shoot and stem hardness, as well as a positive correlation with the carbohydrate and moisture content of the tested cultivars. These results agree with the reports of Abedi [32], who noted that the population parameters of *Ectomyelois ceratoniae* Zeller (Lepidoptera: Pyralidae) significantly correlated with the primary and secondary metabolites of host plants. Our findings regarding the life table parameters on cultivar CP69-1062 were different from Sedighi’s [35] findings, who reported that *r*, *R*_0_, *GRR,* and *λ* values were high for *S. nonagrioides* reared on this cultivar. This discrepancy may be due to either genetic variations in studied populations or differences in the rearing conditions of the tested insects.

The cluster analysis of the tested sugarcane cultivars indicated that SP70-1143 classified in cluster C was the most resistant cultivar. Cluster B consisted of cultivars CP48-103 and CP69-1062 as susceptible cultivars, and other cultivars classified in cluster A were partially resistant cultivars. The *S. nonagrioides* reared on cultivars CP48-103 and CP69-1062 showed higher immature survival and fecundity than that reared on other cultivars, which led to the significant increase in the values of *r*, *R*_0_, and *λ* on these cultivars. In addition, the low values of *r*, *R*_0_, and *λ* in *S. nonagrioides* fed on cultivar SP70-1143 indicated that this cultivar is an unsuitable host for the population increase of this pest. Hence, it could be recommended for cultivation in sugarcane fields where the risk of *S. nonagrioides* damage is high.

## 5. Conclusions

The results of this study confirm that the quality of sugarcane cultivars influenced the developmental rate and population parameters of *S. nonagrioides.* The *S. nonagrioides* reared on cultivars CP48-103 and CP69-1062 showed faster development and higher survival and fecundity than that reared on the other cultivars, leading to the highest intrinsic rate of increase. Therefore, these can be considered as the most suitable cultivars for *S. nonagrioides*, which would result in higher infestations in the sugarcane field [87]. However, cultivar SP70-1143 was the least suitable (most resistant) cultivar for *S. nonagrioides*, which could be recommended for cultivation in the infested sugarcane farms, where the damage of *S. nonagrioides* is usually high. Since CP57-614, CP73-21, and SP70-1143 are reported as resistant sugarcane cultivars to *S. cretica* [77], they can be recommended for use in transgenic expression for resistance to *S. nonagrioides* and *S. cretica*. The outcomes obtained in this research may improve the use of resistant cultivars in the better management of sugarcane stem borers in IPM programs, which, in turn, lead to the reduction in the loss caused by this pest in sugarcane fields. Since volatiles and secondary chemicals derived from non-living plants could be different from those emitted by living plants, to generalize the results of this study to field conditions, further works about the oviposition preference and population parameters of *S. nonagrioides* on living sugarcane cultivars will be necessary.

## Figures and Tables

**Table 1:** Mean (±SE) physical and biochemical traits (n = 5) in examined sugarcane cultivars.

Parameters	Cultivars					
	CP48-103	IRC99-02	CP69-1062	CP73-21	CP57-614	SP70-1143
Moisture content (%)	83.27 ± 0.20 b	86.13 ± 0.57 a	87.80 ± 0.16 a	77.92 ± 0.37 c	71.96 ± 0.57 d	66.77 ± 0.58 e
Trichome density (per cm^2^)	1.40 ± 0.25 d	1.20 ± 0.2 d	1.40 ± 0.25 d	5.80 ± 0.20 c	9.20 ± 0.37 b	12.60 ± 0.40 a
Shoot rind hardness (Newton)	14.80 ± 0.37 f	17.80 ± 0.40 e	22.0 ± 0.32 d	32.60 ± 0.25 c	36.20 ± 0.49 b	46.40 ± 0.25 a
Stem rind hardness (Newton)	56.80 ± 0.58 f	86.80 ± 0.37 e	111.80 ± 0.58 d	120.0 ± 0.45 c	140.0 ± 0.45 b	163.0 ± 0.45 a
Stem pith hardness (Newton)	10.20 ± 0.37 c	9.00 ± 0.32 c	8.80 ± 0.20 c	19.0 ± 0.37 b	32.60 ± 0.25 a	33.20 ± 0.37 a
Total protein (mg/mL)	0.015 ± 0.002 d	0.069 ± 0.004 b	0.014 ± 0.002 d	0.048 ± 0.002 c	0.055 ± 0.004 c	0.096 ± 0.002 a
Carbohydrate content (mg/mL)	396.78 ± 1.30 b	394.36 ± 0.62 b	430.79 ± 1.48 a	394.29 ± 0.93 b	398.19 ± 1.15 b	399.67 ± 0.97 b
Total tannins (mg CE/100 g FW)	23.36 ± 0.32 e	31.664 ± 0.81 c	28.490 ± 0.44 d	30.988 ± 0.52 c	33.882 ± 0.23 b	37.972 ± 0.51 a
Total phenolics (mg GA/100 g FW)	8.374 ± 0.15 e	10.966 ± 0.17 c	9.69 ± 0.03 d	10.833 ± 0.17 c	12.726 ± 0.16 b	13.862 ± 0.12 a
Flavonoids content(mg QE/g FW)	58.57 ± 0.36 e	147.44 ± 3.55 b	108.68 ± 2.36 d	122.75 ± 1.49 c	174.97 ± 1.95 b	256.94 ± 2.64 a

Means in each row followed by different letters are significantly different (*p* < 0.05, Tukey’s HSD test).

**Table 2:** Mean (±SE) developmental time (day) and survival rate (%) of *Sesamia nonagrioides* immature stages on examined sugarcane cultivars.

Cultivars	Egg Incubation	Larval Period	Pre-Pupal Period	Pupal Period	Developmental Time	Immature Survival
CP48-103	6.01 ± 0.01 b	31.63 ± 1.02 e	2.03 ± 0.01 a	10.66 ± 0.05 cd	50.33 ± 1.07 e	81.25 ± 3.07 a
IRC99-02	6.04 ± 0.01 ab	33.00 ± 0.73 de	2.04 ± 0.02 a	10.70 ± 0.08 cd	51.68 ± 0.75 de	55.61 ± 3.93 b
CP69-1062	6.06 ± 0.02 a	34.26 ± 0.51 d	2.03 ± 0.01 a	10.52 ± 0.05 d	52.73 ± 0.53 d	76.25 ± 3.36 a
CP73-21	6.06 ± 0.02 a	36.70 ± 0.51 c	2.05 ± 0.02 a	10.73 ± 0.08 bc	55.38 ± 0.55 c	58.13 ± 3.89 b
CP57-614	6.07 ± 0.02 a	40.79 ± 0.88 b	2.09 ± 0.03 a	10.96 ± 0.08 b	60.15 ± 0.99 b	48.13 ± 3.95 b
SP70-1143	6.10 ± 0.03 a	50.09 ± 2.12 a	2.09 ± 0.04 a	11.19 ± 0.07 a	75.81 ± 2.19 a	19.34 ± 2.56 c

Means in each column followed by different letters are significantly different (*p* < 0.05, paired bootstrap test).

**Table 3:** Mean (±SE) duration (day) of pre-oviposition and oviposition period, fecundity (eggs), fertility (percentage of hatched eggs), and adult longevity (day) of *Sesamia nonagrioides* reared on examined sugarcane cultivars.

Cultivars	APOP	TPOP	Oviposition Period	Fecundity (Eggs/Female)	Fertility (%)	Male Adult Longevity	Female Adult Longevity
CP48-103	0.97 ± 0.02 b	52.69 ± 1.52 d	4.70 ± 0.12 a	228.79 ± 7.12 a	94.71 ± 0.16 a	6.88 ± 0.13 a	6.04 ± 0.12 a
IRC99-02	1.00 ± 0.01 b	54.64 ± 1.15 dc	3.56 ± 0.10 c	188.60 ± 6.81 c	88.25 ± 0.21 c	6.60 ± 0.13 ab	4.84 ± 0.12 b
CP69-1062	0.85 ± 0.04 c	54.00 ± 0.79 d	4.79 ± 0.12 a	228.76 ± 7.17 a	93.50 ± 0.14 a	6.57 ± 0.14 ab	5.86 ± 0.12 a
CP73-21	1.00 ± 0.01 b	57.18 ± 0.71 bc	3.79 ± 0.11 c	213.88 ± 7.72 ab	89.62 ± 0.19 b	6.44 ± 0.14 b	4.93 ± 0.13 b
CP57-614	0.97 ± 0.02 b	60.50 ± 1.54 b	4.14 ± 0.10 b	197.38 ± 11.15 bc	82.89 ± 0.14 d	6.64 ± 0.08 ab	5.05 ± 0.14 b
SP70-1143	1.19 ± 0.08 a	78.89 ± 2.97 a	2.95 ± 0.16 d	67.76 ± 9.09 d	74.75 ± 0.72 e	5.99 ± 0.12 c	4.61 ± 0.10 c

APOP, adult pre-ovipositional period (from the emergence of an adult female to the start of its oviposition); TPOP, total pre-ovipositional period (from egg to first oviposition). Means in each column followed by different letters are significantly different (*p* < 0.05, paired bootstrap test).

**Table 4:** Age-stage, two-sex life table parameters (mean ± SE) of *Sesamia nonagrioides* reared on examined sugarcane cultivars.

Cultivars	*GRR* (offspring)	*R*_0_ (offspring)	*r* (day^−1^)	*λ* (day^−1^)	*T* (day)
CP48-103	388.90 ± 61.86 ab	99.76 ± 9.74 a	0.0977 ± 0.0036 a	1.1027 ± 0.0040 a	47.06 ± 1.17 e
IRC99-02	230.55 ± 40.67 c	45.97 ± 6.62 b	0.0706 ± 0.0034 c	1.0731 ± 0.0037 c	54.11 ± 1.35 d
CP69-1062	478.28 ± 79.19 a	90.10 ± 9.28 a	0.0818 ± 0.0021 b	1.0853 ± 0.0023 b	54.91 ± 0.77 d
CP73-21	209.08 ± 34.27 c	58.81 ± 7.85 ab	0.0697 ± 0.0025 c	1.0722 ± 0.0027 c	58.25 ± 0.79 c
CP57-614	256.23 ± 52.24 bc	44.41 ± 6.97 b	0.0607 ± 0.0028 d	1.0626 ± 0.0030 d	62.21 ± 1.51 b
SP70-1143	83.06 ± 37.00 d	5.98 ± 1.46 c	0.0212 ± 0.0030 e	1.0215 ± 0.0031 e	82.63 ± 3.36 a

Means in each column followed by different letters are significantly different (*p* < 0.05, paired bootstrap test). *GRR*, gross reproductive rate; *R*_0_, net reproductive rate; *r*, intrinsic rate of increase; *λ*, finite rate of increase; *T*, mean generation time.

**Table 5:** Pearson correlation coefficients (*r*) of population parameters and oviposition preference of *Sesamia nonagrioides* reared on examined sugarcane cultivars with some biochemical and physical traits of these cultivars.

Parameters	Total Protein	Carbohydrate Content	Condensed Tannins	Total Phenolics	Flavonoids Content	Moisture Content	Trichome Density	Shoot Rind Hardness	Stem Rind Hardness	Stem Silica	Shoot Silica
Developmental time	0.776	−0.137	0.841 *	0.870 *	0.920 **	−0.892 *	0.921 **	0.915 **	0.852 *	−0.117	−0.220
Survival rate	−0.953 **	0.354	−0.954 **	−0.961 **	−0.978 **	0.848 *	−0.881 *	−0.873 *	−0.828 *	0.471	0.470
Fecundity	−0.872 *	0.233	−0.814 *	−0.813 *	−0.914 **	0.754	−0.770	−0.755	−0.690	0.349	0.348
Weight of female pupa	−0.921 **	0.554	−0.829 *	−0.852 *	−0.866 *	0.874 *	−0.864 *	−0.829 *	−0.693	0.511	0.629
Weight of male pupa	−0.872 *	0.458	−0.817 *	−0.868 *	−0.890 *	0.926 **	−0.910 *	−0.855 *	−0.725	0.358	0.533
Female longevity	−0.941 **	0.518	−0.890 *	−0.854 *	−0.821 *	0.628	−0.671	−0.694	−0.670	0.762	0.633
Male longevity	−0.776	0.016	−0.842 *	−0.783	−0.867 *	0.667	0.747	−0.831 *	−0.825 *	0.242	0.117
Oviposition preference	−0.845 *	0.486	−0.898 *	−0.927 **	−0.843 *	0.876 *	−0.904 *	−0.897 *	−0.832 *	0.463	0.554
*R*_0_	−0.983 **	0.405	−0.961 **	−0.945 **	−0.961 **	0.778	−0.815 *	−0.814 *	−0.783	0.345	0.534
*r*	−0.902 **	0.192	−0.954 **	−0.947 **	−0.986 **	0.826 *	−0.880 *	−0.899 *	−0.880 *	0.594	0.310
*λ*	−0.904 **	0.193	−0.958 **	−0.950 **	−0.987 **	0.824 *	−0.879 *	−0.901 *	−0.882 *	0.353	0.312
*T*	0.815 *	−0.094	0.900 *	0.903 *	0.953 **	−0.847 *	0.900 *	0.924 **	0.895 *	−0.180	−0.194

* and ** show significant correlations at *p* < 0.05 and *p* < 0.01, respectively. *R*_0_, net reproductive rate; *r*, intrinsic rate of increase; *λ*, finite rate of increase; *T*, mean generation time.

**Table 6:** Pearson correlation coefficients (*r*) between population parameters and pupal weight of *Sesamia nonagrioides* reared on examined sugarcane cultivars.

Parameters	Intrinsic Rate of Increase		Net Reproductive Rate		Finite Rate of Increase		Mean Generation Time		Fecundity		Female Longevity		Male Longevity	
	*r*	*p*	*r*	*p*	*r*	*p*	*r*	*p*	*r*	*p*	*r*	*p*	*r*	*p*
Weight of female pupa	0.910	0.012	0.926	0.008	0.908	0.012	−0.877	0.022	0.901	0.014	0.843	0.035	-	-
Weight of male pupa	0.921	0.009	0.897	0.015	0.918	0.01	−0.914	0.011	0.93	0.007	-	-	0.788	0.063

## Data Availability

The data presented in this study are available on request from the corresponding author.
